# Toward reaching hepatitis B goals: hepatitis B epidemiology and the impact of two decades of vaccination, Georgia, 2021

**DOI:** 10.2807/1560-7917.ES.2023.28.30.2200837

**Published:** 2023-07-27

**Authors:** Nino Khetsuriani, Amiran Gamkrelidze, Shaun Shadaker, Maia Tsereteli, Maia Alkhazashvili, Nazibrola Chitadze, Irina Tskhomelidze, Lia Gvinjilia, Francisco Averhoff, Gavin Cloherty, Qian An, Giorgi Chakhunashvili, Jan Drobeniuc, Paata Imnadze, Khatuna Zakhashvili, Paige A Armstrong

**Affiliations:** 1 Global Immunization Division, Centers for Disease Control and Prevention (CDC), Atlanta, United States; 2 National Center for Disease Control and Public Health of Georgia (NCDC), Tbilisi, Georgia; 3 Division of Viral Hepatitis, Centers for Disease Control and Prevention (CDC), Atlanta, United States; 4 The Task Force for Global Health, Tbilisi, Georgia; 5 Eastern Europe and Central Asia Regional Office, Centers for Disease Control and Prevention (CDC), Tbilisi, Georgia; 6 Abbott Diagnostics, Abbott Park, IL, United States; 7 Abbott Pandemic Defense Coalition, Abbott Park, IL, United States

**Keywords:** Georgia, Hepatitis B, serosurvey, prevalence, Hepatitis B vaccine

## Abstract

**Background:**

Georgia has adopted the World Health Organization European Region’s and global goals to eliminate viral hepatitis. A nationwide serosurvey among adults in 2015 showed 2.9% prevalence for hepatitis B virus (HBV) surface antigen (HBsAg) and 25.9% for antibodies against HBV core antigen (anti-HBc). HBV infection prevalence among children had previously not been assessed.

**Aim:**

We aimed to assess HBV infection prevalence among children and update estimates for adults in Georgia.

**Methods:**

This nationwide cross-sectional serosurvey conducted in 2021 among persons aged ≥ 5 years used multi-stage stratified cluster design. Participants aged 5–20 years were eligible for hepatitis B vaccination as infants. Blood samples were tested for anti-HBc and, if positive, for HBsAg. Weighted proportions and 95% confidence intervals (CI) were calculated for both markers.

**Results:**

Among 5–17 year-olds (n = 1,473), 0.03% (95% CI: 0–0.19) were HBsAg-positive and 0.7% (95% CI: 0.3–1.6) were anti-HBc-positive. Among adults (n = 7,237), 2.7% (95% CI: 2.3–3.4) were HBsAg-positive and 21.7% (95% CI: 20.4–23.2) anti-HBc-positive; HBsAg prevalence was lowest (0.2%; 95% CI: 0.0–1.5) among 18–23-year-olds and highest (8.6%; 95% CI: 6.1–12.1) among 35–39-year-olds.

**Conclusions:**

Hepatitis B vaccination in Georgia had remarkable impact. In 2021, HBsAg prevalence among children was well below the 0.5% hepatitis B control target of the European Region and met the ≤ 0.1% HBsAg seroprevalence target for elimination of mother-to-child transmission of HBV. Chronic HBV infection remains a problem among adults born before vaccine introduction. Screening, treatment and preventive interventions among adults, and sustained high immunisation coverage among children, can help eliminate hepatitis B in Georgia by 2030.

**Key public health message**

**What did you want to address in this study?**

The country of Georgia has adopted the World Health Organization European Region’s and global goals to eliminate viral hepatitis. In 2015, 2.9% of adults in Georgia were chronically infected with hepatitis B virus (HBV). Data on the frequency of chronic HBV infection among children were not available. In 2021, we conducted a national survey to determine frequency of HBV infection among children and adults in Georgia.

**What have we learnt from this study?**

In 2021, only very few children (0.03%) in Georgia had chronic HBV infection. The low level of HBV infection among children results from high coverage with infant hepatitis B vaccination introduced in 2001. With 2.7% of adults (estimated 77,000 persons) infected in 2021, chronic HBV infection remains a problem among those born before hepatitis B vaccine introduction.

**What are the implications of your findings for public health?**

Georgia has achieved the European Region’s hepatitis B control goal and has probably achieved the elimination of mother-to-child transmission of HBV. Near absence of childhood HBV infections will allow to focus on hepatitis B screening, treatment and prevention among adults. High infant immunisation coverage for hepatitis B should also be sustained. These efforts can help achieve the elimination of hepatitis B as a public health threat by 2030 in Georgia.

## Introduction

Georgia, a middle-income member state of the World Health Organization (WHO) European Region (population 3.7 million), has historically had an intermediate level of hepatitis B virus (HBV) endemicity [1]. The estimated prevalence of hepatitis B surface antigen (HBsAg), a marker of chronic/active HBV infection, in Georgia was 5.5% among women of child-bearing age in 2000 [2]. The HBsAg prevalence in surveys focused on high-risk groups was 4.1–4.3% between 1997 and 1998 and 2.8% in 2012 [3-5]. In 2015, a nationwide serosurvey among adults ≥ 18 years showed an HBsAg prevalence of 2.9%, while the prevalence of antibodies against HBV core antigen (anti-HBc), indicating exposure to HBV, was 25.9% [6]. A modelling study estimated HBsAg prevalence in Georgia in 2015 to be 2.5% in the general population and 0.4% among 5-year-olds [7].

**Figure 1:**
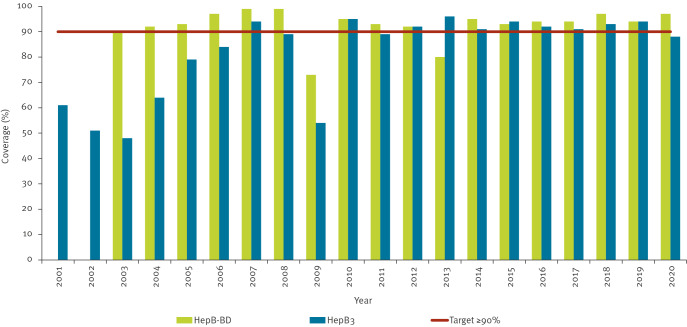
WHO-UNICEF estimates of immunisation coverage with at least three doses of hepatitis B-containing vaccines (HepB3) and with hepatitis B vaccine birth dose (HepB-BD), Georgia, 2001–2020

HepB3: At least three doses of hepatitis B-containing vaccines; HepB-BD: hepatitis B vaccine birth dose; UNICEF: United Nations Children’s Fund; WHO: World Health Organization.

Hepatitis B vaccine, the most efficient and cost-effective means of preventing HBV infection [8], was introduced in Georgia’s national immunisation programme in 2001. The birth dose (HepB-BD) for all newborns, essential for prevention of mother-to-child transmission (MTCT) of HBV, was introduced in 2003. Initially, a total of three doses of hepatitis B vaccine were recommended. Since 2010, the schedule includes four doses (Table 1). Immunisation coverage with three or more doses of hepatitis B-containing vaccines (HepB3) in Georgia has been consistently above 90% since 2012 (Figure 1) [9,10]. Coverage with HepB-BD has been at least 90% except in 2009 (73%) and 2013 (80%), when there were monovalent hepatitis B vaccine stock-outs.

**Table 1:** History of national hepatitis B vaccination schedules in Georgia, 2001–2022

Period	Birth dose^a^	Subsequent doses		Total doses
		Vaccines	Recommended ages	
2001^b^–2002	No	Hepatitis B (paediatric)	2M, 3M, 8M	3
2003–2009	Yes	Hepatitis B (paediatric)	B, 2M, 4M	3
2010–November 2015	Yes	Pentavalent (DTwP-HiB-HepB)	B, 2M, 3M, 4M	4
December 2015–present	Yes	Hexavalent (DTaP-HiB-HepB-IPV)	B, 2M, 3M, 4M	4

B: birth; DTaP: diphtheria and tetanus toxoid and acellular pertussis; DTwP: diphtheria and tetanus toxoid and whole cell pertussis; HepB: hepatitis B; HiB: *Haemophilus influenzae* type B; IPV: inactivated polio vaccine; M: months.

^a^ Monovalent hepatitis B vaccine (paediatric formulation); recommended for all newborns within the first 12 h of birth.

^b^ Hepatitis B vaccine was introduced in 2000 in selected regions.

Target for validation of the achievement of the WHO European Regional hepatitis B control goals is ≥ 90% [10]. HepB3 reflects coverage with three doses of hepatitis B-containing vaccine during the period 2001 through 2009, and with four doses since 2010. Source: [9].

Additional measures to prevent MTCT of HBV initiated in 2006 included hepatitis B screening of pregnant women and administration of hepatitis B immunoglobulin (HBIG) to infants born to infected mothers. The HBsAg positivity among pregnant women screened between 2006 and 2011 was 3% [11]. According to unpublished data from the National Center for Disease Control and Public Health of Georgia (NCDC), 94–99% of pregnant women were screened annually from 2018 through 2020 and 1.2–1.4% of them were HBsAg-positive. During this period, at least 82% of eligible infants received HBIG.

Among adults, hepatitis B vaccination has been mandatory for some occupational groups, including healthcare workers, since 2019 [12] and is offered to persons with hepatitis C through the National Hepatitis C Elimination Programme since 2015. However, data on the uptake of hepatitis B vaccination in these groups are unavailable.

Georgia has adopted WHO global and European Regional goals for viral hepatitis. The WHO European Region goal of hepatitis B control requires an HBsAg prevalence lower than 0.5% in vaccinated age groups, along with at least 90% coverage with HepB3 and measures to prevent MTCT of HBV [10,13]. The WHO has established goals for the elimination of viral hepatitis as a public health threat by 2030 [14], including elimination of MTCT of HBV, which requires an HBsAg prevalence of no more than 0.1% among children and at least 90% coverage with HepB-BD and HepB3 [15]. In addition, combating viral hepatitis is part of the Sustainable Development Goals Target 3.3 [16]. 

The impact of hepatitis B vaccination on the prevalence of HBV infection in Georgia had not previously been assessed. Considering the progress made towards the national hepatitis C elimination goal adopted in 2015 [17,18] and substantial burden of HBV infection, elimination of hepatitis B was included in the 2021–2025 Strategic Plan for the Elimination of Hepatitis in Georgia. Because of the early initiation of hepatitis C elimination efforts, Georgia’s hepatitis elimination programme is further advanced than in other intermediate and high-endemicity countries in the Caucasus, Eastern Europe and Central Asia which also have a substantial hepatitis B disease burden [19-27].

To assess prevalence of HBV infection among children, update the estimates for adults, evaluate progress towards reaching elimination targets, and better understand the epidemiology of HBV infections in Georgia, we conducted a nationwide serosurvey in 2021. The hepatitis C component of the serosurvey has been published separately [28].

## Methods

### Survey design

We conducted a cross-sectional household survey using a stratified, multi-stage cluster design. The serosurvey was designed to provide national and regional seroprevalence estimates of HBV, as well as hepatitis C virus (HCV) and SARS-CoV-2 infections in Georgia among children (5–17 years) and adults (≥ 18 years). Participant enrolment took place during the period from June to October 2021. A detailed description of the survey methodology is appended in the Supplement. Briefly, the sampling frame was the general population of Georgia aged ≥ 5 years. Required sample size was estimated, based on the needs of the SARS-CoV-2 component, as 8,010 for adults and 2,320 for children. This provided sufficient sample size for hepatitis B and C components as well. A total of 267 clusters, defined as census enumeration areas, were selected with probability proportionate to size and allocated across 10 strata throughout Georgia. Within each cluster, households for enrolment were chosen systematically, using a skip pattern. Individual participants were selected using Kish tables. We selected one adult aged ≥ 18 years per household. In households with at least one child of eligible age, we also selected one child aged 5–17 years per household. 

### Survey questionnaire 

The survey questionnaire was administered by trained field staff in face-to-face interviews with participants or their parents or caregivers (for children). The responses were recorded electronically using tablets and uploaded to a cloud-based server (ODK Inc.). A survey questionnaire included demographic data, clinical and behavioural history, immunisation status, and hepatitis B-related awareness and practices. Participants aged 5–20 years had been eligible for routine hepatitis B vaccination as infants.

### Laboratory testing

Whole venous blood (ca 10 mL) was collected from the participants. Serum was separated on site and transported under cold chain to Tbilisi for testing at the Serology Laboratory, Lugar Center, NCDC. All specimens were tested for anti-HBc, and anti-HBc-positive samples were also tested for HBsAg; HBsAg-positive samples were tested for HBV DNA and viral loads were quantified. Laboratory testing was conducted using the Abbott ARCHITECT i2000SR system (Abbott Diagnostics). Chemiluminescence assays ARCHITECT Anti-HBc II and ARCHITECT HBs Ag Qualitative A II were used for detection of anti-HBc and HBsAg, respectively. Real-rime PCR with Abbott Real*Time* HBV (m2000rt) (Abbott Molecular Inc.) was used to detect HBV DNA.

Anti-HBc-negative persons were considered unexposed/uninfected. Among anti-HBc-positive people, HBsAg-positive participants were considered as having chronic HBV infection (the probability of encountering active acute HBV infection in a cross-sectional survey in a low incidence setting is very small), while anti-HBc-positive/HBsAg-negative persons were considered as having resolved HBV infection [29]. The HBV viral load levels were categorised as < 2,000 IU/mL, 2,000–19,000 IU/mL and ≥ 20,000 IU/mL [30].

### Statistical analysis 

In statistical analysis, primary outcome measures for seroprevalence were adjusted proportions with 95% confidence intervals (CI). Thus, all percentages and CI reported below are adjusted. To produce nationally representative estimates of seroprevalence, results were weighted at cluster, household and individual levels, and estimates were adjusted by sex (collected as a binary variable), age and geographical distribution using 2014 census data.

Potential risk factors for anti-HBc positivity (i.e. for acquiring HBV infection) were analysed in bivariate and multivariate analyses. Chi-squared test was used in bivariate analysis, with p values < 0.05 considered significant. Variables associated with anti-HBc positivity in bivariate analysis were included in the multivariable regression model and adjusted odds ratios (OR) and 95% CIs were calculated.

For comparisons of prevalence estimates for adult participants with those from the 2015 serosurvey which had comparable design [6], ages of participants of the 2015 serosurvey were adjusted to their ages in 2021 as described in the Supplement. All analysis was performed in SAS version 9.4.

## Results

A total of 8,710 individuals aged ≥ 5 years were enrolled, including 7,237 adults aged ≥ 18 years (90.3% participation rate) and 1,473 children aged 5–17 years (72.2% participation rate; eligible children in the enrolled households: n = 2,041). Among all participants of the serosurvey, the weighted prevalence of anti-HBc was 17.9% (95% CI: 16.7–19.1). The weighted prevalence of HBsAg was 2.2% (95% CI: 1.8–2.8).

### Prevalence among children

Among the 1,473 child participants, seven tested positive for anti-HBc, for a weighted prevalence of 0.7% (95% CI: 0.3–1.6). Only one child was also HBsAg-positive, for a weighted prevalence of 0.03% (95% CI: 0–0.19).

There was no geographical clustering of anti-HBc-positive children; two of them were male and five were female; three were aged 5–9 years and four were aged 10–17 years. Five anti-HBc-positive children had received three doses of hepatitis B vaccine and two had received one dose. Five anti-HBc-positive children had received HepB-BD, which had been given timely to all of them.

The HBsAg-positive child was a 7-year-old female who received a single dose of hepatitis B vaccine at 2 months of age. She was born in 2013, when Georgia experienced a shortage of monovalent hepatitis B vaccine. The adult participant from the same household, a female in her 40s, was also HBsAg-positive; both had low viral loads (child: 30 IU/mL; adult: 40 IU/mL).

### Prevalence among adults

**Figure 2:**
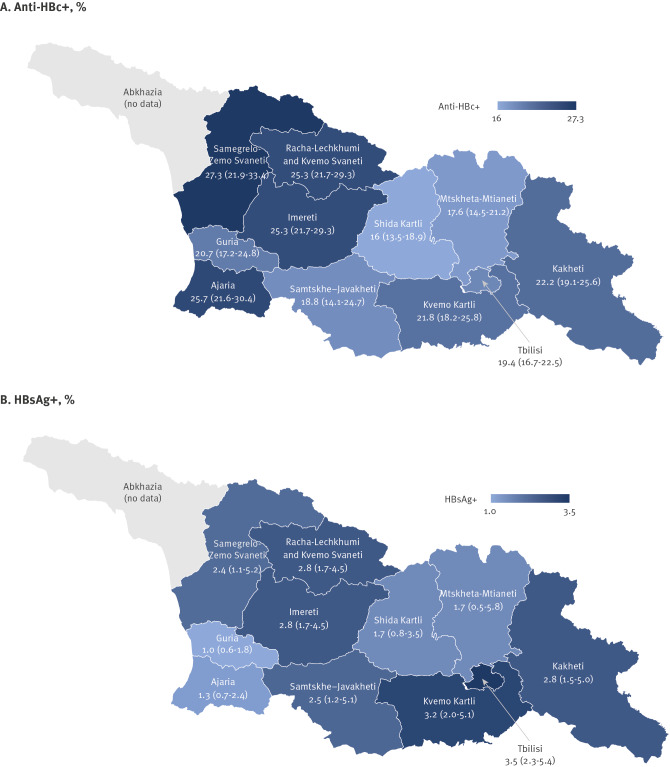
Prevalence of anti-HBc and HBsAg, by region, nationwide serosurvey, Georgia, 2021 (n = 8,710)

Anti-HBc: antibodies against hepatitis B virus core antigen; HBsAg: hepatitis B virus surface antigen.

Of 7,237 adult participants tested, 1,670 were anti-HBc-positive and 162 were HBsAg-positive, for a weighted prevalence of 21.7% (95% CI: 20.4–23.2) for anti-HBc and 2.7% (95% CI: 2.2–3.4) for HBsAg (Table 2). Anti-HBc prevalence increased with age from 1.3% (95% CI: 0.6–3.1) among 18–23-year-olds to 11.7% (95% CI: 8.2–16.5) among 30–34-year-olds, then plateaued, with point estimates between 27.4% and 28.6% in older age groups (Table 2). The HBsAg prevalence increased from 0.2% (95% CI: 0.0–1.5) among 18–23-year-olds to 8.6% (95% CI: 6.1–12.1) among 35–39-year-olds, but declined in older age groups, reaching 1.7% (95% CI: 1.2–2.6) among persons aged ≥ 60 years (Table 2). Anti-HBc prevalence did not differ by sex, but HBsAg prevalence was higher among males than females (3.6%; 95% CI: 2.8–4.7 vs 2.0%; 95% CI: 1.4–2.7; p = 0.003). Persons with higher education and income levels had significantly lower anti-HBc prevalence than those with less education and lower incomes (p < 0.0001 for both). Persons who were unemployed had higher prevalence of HBsAg (p = 0.03) (Table 2). Differences by region were significant for anti-HBc (p < 0.001) but not for HBsAg (p = 0.23). Anti-HBc prevalence ranged between 16.0% and 27.3% and was highest (> 25%) in Samegrelo-Zemo Svaneti, Adjara and Imereti regions and lowest (< 20%) in Shida Kartli, Mtskheta-Mtianeti and Samtskhe-Javakheti regions (Figure 2).

**Table 2:** Prevalence of anti-HBc and HBsAg by demographic characteristics, nationwide serosurvey, Georgia, 2021 (n = 8,710)

Characteristics	Total	Anti-HBc-positive				HBsAg-positive			
		n	Weighted %	Adjusted 95% CI	p value	n	Weighted %	Adjusted 95% CI	p value
All ages ≥ 5 years	8,710	1,677	17.9	16.7–19.1	NA	163	2.2	1.8–2.8	NA
Children 5–17 years	1,473	7	0.7	0.3–1.6	NA	1	0.03	0–0.19	NA
Adults ≥ 18 years	7,237	1,670	21.7	20.4–23.2	NA	162	2.7	2.2–3.4	NA
Age groups in years^a^									
18–23	322	6	1.3	0.6–3.1	< 0.0001	1	0.2	0.0–1.5	< 0.0001
24–29	430	15	3.8	2.0–7.1		3	0.9	0.2–4.8	
30–34	565	62	11.7	8.2–16.5		14	2.9	1.2–7.0	
35–39	684	179	27.8	23.4–32.6		46	8.6	6.1–12.1	
40–49	1,233	319	27.4	23.9–31.3		36	3.0	2.0–4.5	
50–59	1,517	402	28.2	25.0–31.7		25	3.0	1.6–5.7	
≥ 60	2,476	687	28.6	26.1–31.2		36	1.7	1.2–2.6	
Sex (binary)^a^									
Male	2,409	618	23.1	20.9–25.5	0.05	77	3.6	2.8–4.7	0.003
Female	4,828	1,052	20.5	19.1–22.1		85	2.0	1.4–2.7	
Ethnicity^a^									
Georgian	6,407	1,492	21.6	20.1–23.1	0.41	141	2.7	2.2–3.4	0.16
Armenian	353	57	18.9	13.6–25.7		2	0.5	0.1–2.2	
Azerbaijani	280	75	23.9	17.2–32.1		12	3.9	2.0–7.5	
Other	133	33	30.4	17.3–47.9		4	2.5	0.8–7.8	
Highest level of school completed^a^									
Primary or less	645	182	28.4	23.8–34.4	< 0.001	15	2.6	1.1–5.9	0.64
Secondary	2,355	564	22.2	20.0–24.5		47	2.5	1.7–3.5	
Vocational	1,465	377	27.3	24.1–30.8		39	3.6	2.2–5.8	
University or higher	2,704	533	17.8	16.1–19.7		59	2.6	1.8–3.7	
Employment status^a^									
Unemployed	1,263	313	23.8	20.7–27.2	0.17	37	4.3	2.6–7.1	0.03
Other^b^	5,904	1,342	21.3	19.8–22.9		122	2.3	1.8–2.9	
Monthly household income in GEL (EUR)^a,c^									
≤ 500 (≤ 136)	2,362	619	26.2	24.0–28.6	< 0.0001	44	2.4	1.5–3.7	0.81
501–2,000 (137–545)	3,605	786	20.7	19.0–22.6		88	2.7	2.0–3.5	
> 2,000 (≥ 545)	192	29	12.5	7.9–19.1		3	1.9	0.5–6.6	

Anti-HBc: antibodies against hepatitis B virus core antigen; CI: confidence intervals; EUR: Euro; GEL: Georgian lari; HBsAg: hepatitis B virus surface antigen; NA: not applicable.

^a^ Only adult participants are included.

^b^ Employed, student, homemaker and retired.

^c^ Average exchange rate during the survey data collection period: 1 EUR = 3.67 GEL; source: https://nbg.gov.ge/en/monetary-policy/currency.

Totals vary as not all participants answered all questions: age reported for 7,227 participants, ethnicity for 7,173, education level for 7,169, employment status for 7,167 and monthly income for 6,159 participants.

The estimates for Imereti region are applied to the Racha-Lechkhumi and Kvemo Svaneti region which, due to its small population (n = 30,778), was combined with Imereti in one stratum.

**Figure 3:**
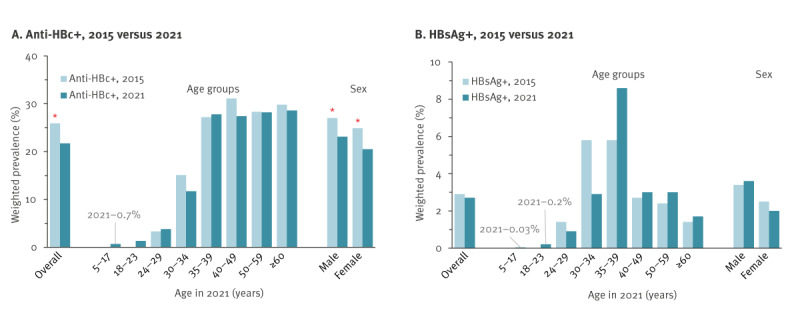
Anti-HBc and HBsAg seroprevalence in Georgia, 2015 vs 2021

Anti-HBc: antibodies against hepatitis B virus core antigen; HBsAg: hepatitis B virus surface antigen.

Overall, the anti-HBc prevalence among adults ≥ 18 years was lower in 2021 than in 2015 (21.7%; 95% CI: 20.4–23.2 vs 25.9%; 95% CI: 24.1–27.6; p = 0.0004). Comparison with age-adjusted estimates from 2015 revealed no significant differences in anti-HBc seroprevalence for those aged ≥ 24 years, included in both surveys (Figure 3). Anti-HBc prevalence among adults aged 18–23 years in 2021, who were children at the time of the previous survey and therefore not included there, was very low (1.3%; 95% CI: 0.6–1.3) (Table 2). The lower anti-HBc prevalence in 2021 vs 2015 was significant for both males (p = 0.02) and females (p = 0.0006). No significant differences between the two surveys were observed for HBsAg prevalence (Figure 3).

The data set from the 2015 sersosurvey was also used for this analysis [6]. Ages for participants of 2015 serosurvey were adjusted (added 6 years) to reflect their age in 2021. Only the data from 2021 are presented for ages < 24 years because the serosurvey in 2015 only included persons aged 18 years or older at the time (24 years or older in 2021). The p values comparing anti-HBc-positives in 2021 vs 2015 were: overall: 0.0004; males: 0.02; females: 0.0006; for all other comparisons: > 0.05. Asterisks indicate the comparisons with p values < 0.05.

Extrapolation of the survey estimates to respective population data [31] resulted in an estimated 200 children (range: 0–1,200) and 77,000 (range: 62,000–97,000) adults living with chronic HBV infection in Georgia in 2021. This was comparable to the estimate of adults living with chronic HBV infection in 2015 (ca 80,000) [6].

### HBV viral load among adults

We detected HBV DNA in 138 (90.7%) of 162 HBsAg-positive adult samples. Of those, 96 (65.7%) had viral load < 2,000 IU/mL; 31 (17.9%) had viral load 2,000–19,999 IU/mL, and viral loads ≥ 20,000 IU/mL were observed in 11 participants (7.1%). Males were more likely to have high viral loads than females (p = 0.0008), accounting, in weighted analysis, for 96.9% of those with ≥ 20,000 IU/mL viral load, 67.1% of those with 2,000–19,999 IU/mL and 56.6% of those with < 2,000 IU/mL.

### Risk factors for anti-HBc positivity among adults

Prevalence of selected medical and behavioural risk factors reported by adult participants is given in Table 3. In bivariate analysis, anti-HBc positivity was associated with history of injecting drugs, incarceration, invasive medical procedures or surgery, higher lifetime number of sexual partners, inconsistent condom use and lack of hepatitis B vaccination (p < 0.05 for all; Table 3). In multivariate analysis, only history of injecting drugs (adjusted OR: 5.51; 95% CI: 2.20–13.80) and inconsistent condom use (adjusted OR: 1.84; 95% CI: 1.02–3.32) remained associated with anti-HBc positivity (Table 3).

**Table 3:** Selected risk factors for hepatitis B virus infection reported by adult participants (age ≥ 18 years) and their association with anti-HBc positivity, nationwide serosurvey, Georgia, 2021

Variables	n^a^	Weighted %	Weighted 95% CI	Bivariate analysis, p value	Multivariate analysis	
					Adjusted OR (95% CI)	p value
Healthcare-related factors						
Invasive medical procedure, ever	634	10.8	9.6–12.0	0.02	0.89 (0.67–1.18)^b^	0.41
Surgery, ever	4,061	55.4	53.3–57.4	0.04	0.97 (0.81–1.15)^b^	0.71
Invasive dental procedure, ever	6,713	92.1	90.9–93.2	> 0.05	NA	
Blood transfusion, ever	347	4.7	3.9–5.5	> 0.05	NA	
Dialysis, ever	9	0.1	0.0–0.3	> 0.05	NA	
Last injection given by						
*Doctor or nurse*	3,088	54.7	52.2–57.2	> 0.05	NA	
*Dentist*	1,714	30.3	28.0–32.8			
*Non-healthcare worker*	694	11.5	10.2–13.1			
*Self*	204	3.4	2.8–4.2			
Received hepatitis B vaccine, ≥ 1 doses	83	1.7	1.3–2.2	0.001	1.35 (0.63–2.89)^c^	0.45
Occupational history						
Being a healthcare worker (past or present)	455	5.8	5.0–6.7	> 0.05	NA	
History of incarceration						
Incarcerated, ever	169	3.9	3.3–4.8	0.004	0.90 (0.38–2.16)^b^	0.82
Behavioural factors						
Injected drugs, ever	120	3	2.3–3.9	< 0.0001	5.51 (2.20–13.80)^b^	0.0003
Sex partners, lifetime number						
*0*	477	9.4	8.3–10.8	< 0.0001	Reference group	1
*1–5*	4,824	78.2	76.3–80.1		1.06 (0.54–2.06)	0.87
*> 5*	417	12.3	10.8–14.0		1.00 (0.48, 2.08)	1
Condom use						
*Always*	378	10.6	9.1–12.4	< 0.0001	Reference group	1
*Sometimes/often*	2,200	40.8	38.1–43.6		1.84 (1.02–3.32)	0.04
*Never*	3,154	48.5	45.6–51.5		1.66 (0.94–2.94)	0.08
Men having sex with men, ever						
*Yes*	2	0.1	0.0–0.4	> 0.05	NA	
*No*	2,268	95.9	94.1–97.2			
*Refused to answer*	75	4	2.8–5.8			
Sex with a commercial sex worker, ever	302	6.2	5.1–7.5	> 0.05	NA	

Anti-HBc: antibodies against hepatitis B virus core antigen; CI: confidence intervals; HBsAg: hepatitis B virus surface antigen; NA: not applicable; OR: odds ratio.

^a^ Denominators vary as not all participants answered all questions.

^b^ Reference group: ‘No’.

^c^ Reference group: ‘Yes’.

The multiple regression model included age and all variables with p < 0.05 in bivariate analysis.

### History of hepatitis B vaccination, testing and treatment

Only 83 (adjusted percentage 1.7%; 95% CI: 1.3–2.2) adult participants reported receipt of at least one dose of hepatitis B vaccine. The adjusted proportion of vaccinated persons declined with age from 7.1% (95% CI: 4.3–11.3) among 18–23-year-olds to 0.3% (95% CI: 0.1–0.7) among those aged ≥ 60 years (p < 0.0001). Among 283 current healthcare workers, 31 (14.8%; 95% CI: 9.5–22.5) reported receipt of hepatitis B vaccine.

Among adult participants, 793 (13.5%; 95% CI: 12.1–15.0) reported having previously been tested for hepatitis B and 86, including 53 HBsAg-positive and 33 HBsAg-negative participants, had previously been told by a healthcare provider that they had hepatitis B. Among the 53 HBsAg-positive participants who were aware of their hepatitis B status, 19 (31.1%; 95% CI: 16.0–51.8) reported ever receiving any treatment for hepatitis.

### Hepatitis B-related awareness

Among survey participants, 34.9% (95% CI: 32.6–37.4) had heard of hepatitis B. Among them, 50.1% (95% CI: 46.2–54.1) were aware that HBV infection can be asymptomatic and 46.0% (95% CI: 42.4–49.6) knew that HBV infection could be treated.

Given a list of possible HBV transmission routes, 59.9% (95% CI: 55.9–63.8) among those who had heard of HBV, correctly identified at least one transmission route and gave no incorrect answers, and 17.5% (95% CI: 14.8–20.5) gave a mix of correct and incorrect answers. Most respondents (87.9%; 95% CI: 85.3–90.1) were aware of transmission through blood, followed by sharing needles (45.9%; 95% CI: 40.7–51.0) and sexual contact 45.5%; 95% CI: 41.2–49.9), but only 22.5% (95% CI: 18.9–26.6) knew about perinatal transmission of HBV.

When asked about ways to prevent HBV, 64.1% (95% CI: 60.5–67.4) of participants correctly identified at least one strategy with no incorrect answers, and 7.6% (95% CI: 5.9–9.7) gave a mix of correct and incorrect answers. Among preventive strategies, the awareness was greatest for not sharing needles (45.0%; 95% CI: 40.9–49.3) and avoidance of unsterile/used medical devices (43.8%; 95% CI: 39.9–47.8), followed by vaccination (39.8%; 95% CI: 35.8–44.0), but only 21.6% (95% CI: 18.9–24.7) named condom use. More detailed answers to questions on hepatitis B-related knowledge are appended in the Supplement.

## Discussion

Georgia has been successfully implementing a national hepatitis C elimination programme since 2015 [17,18]. However, testing and treatment options for persons living with chronic HBV infection in Georgia remained limited, and apart from infant vaccination, there have been no national efforts until recently to address hepatitis B burden. This serosurvey provided previously unavailable information to assess the impact of vaccination and help guide implementation of hepatitis B elimination efforts in Georgia following its inclusion in the national strategic plan.

The survey revealed that two decades of successful routine hepatitis B vaccination of infants in Georgia resulted in a very low seroprevalence of HBV infection in vaccinated age groups. The HBsAg prevalence of 0.03% among children is well below the 0.5% European regional hepatitis B control target [10] and meets the ≤ 0.1% seroprevalence target for elimination of MTCT of HBV set forth by the WHO [15]. This finding, in combination with high reported coverage with three doses of hepatitis B-containing vaccines [9], was the basis for validation of Georgia in 2022 by the European Technical Advisory Group of Experts (ETAGE) as having achieved the hepatitis B control [32]. In addition, Georgia is likely to have achieved the elimination of MTCT of HBV and could apply for validation once the procedures and tools of the validation process are finalised [33].

To ensure continued low prevalence in future birth cohorts, high immunisation coverage with hepatitis B-containing vaccines should be maintained. The COVID-19 pandemic resulted in a moderate decline in routine immunisations in Georgia, with 88% coverage with the third dose of hexavalent vaccine in 2020 [9]. Identifying and immunising children born since 2020 who missed their hexavalent vaccine doses would help mitigate the impact of the pandemic and ensure continued high immunisation coverage for hepatitis B [9,34]. High coverage with timely HepB-BD should also be sustained. Further strengthening additional interventions to prevent MTCT, such as antenatal screening for HBV, antiviral treatment of mothers with high viral loads, timely administration of HBIG to all infants born to infected mothers, along with introduction of post-vaccination serology testing of exposed infants, would ensure that babies born to HBV-infected mothers will remain safe from MTCT of the virus [15,30].

In contrast to the very low prevalence among children, hepatitis B remains a problem among adults throughout Georgia, particularly among those born before introduction of routine hepatitis B vaccination. In this survey, HBsAg prevalence among adults was in the intermediate endemicity range, with an estimated 77,000 adults living with chronic HBV infection. There were no significant regional differences in HBsAg prevalence, but the level of previous exposure to HBV infection (anti-HBc-positivity) was highest in western Georgia. While the hepatitis C elimination programme has made demonstrable progress [18,28], the HBsAg prevalence among adults remained unchanged from 2015 [17]. Overall anti-HBc prevalence was slightly lower in 2021, largely due to the low prevalence among those aged 18–23-years. This declining trend is likely to continue as more vaccinated birth cohorts reach adulthood.

The age distribution of anti-HBc positivity suggests that exposure to HBV is rare until ca 30 years of age. This finding is corroborated by the 0.5% HBsAg prevalence among pregnant women younger than 30 years who underwent antenatal screening compared with 1.6% among 30–34-year-olds and 2.6% among 35–44-year-olds (personal communication, Levan Kandelaki, NCDC, 2022). Likely contributors to low HBV seroprevalence among young adults in Georgia include infant immunisations (for some birth cohorts), as well as improvement in infection control and injection safety practices in healthcare settings and improved blood safety in recent years [35-37], along with a shorter cumulative exposure to behavioural risk factors. 

Of note, we observed decline in the HBsAg prevalence among adults after 60 years of age. The decline in HBsAg positivity among the elderly population has been also reported in countries with different levels of HBV endemicity [38-40]. The reasons are not clear and could vary across countries, but in Georgia, where the anti-HBc prevalence plateaued after 35 years of age but HBsAg prevalence declined, the likely explanation would be deaths of HBsAg-positive persons from HBV-related (cirrhosis, liver cancer) or other causes (e.g. high-risk behaviours, such as injectable drug use) resulting in lower HBsAg prevalence in the elderly.

A substantial proportion of unexposed adults born before or shortly after hepatitis B vaccine introduction are unvaccinated and remain susceptible to HBV. The low rates of hepatitis B vaccination among adults in this survey (< 2%), even among healthcare workers (< 15%), suggest a need for additional education and interventions aimed at this group. Compared with HCV infection, which can be effectively treated with a 12-week course of well-tolerated direct acting antivirals in most people, chronic HBV infection requires life-long treatment for those who meet the eligibility criteria [30,41]. Therefore, hepatitis B vaccination is the preferable strategy for reducing hepatitis B infections among adults, especially in high-risk groups (e.g. haemodialysis patients and persons who inject drugs). Expanding adult access to hepatitis B vaccinations, particularly for younger and high-risk groups, along with systematically implementing mandatory hepatitis B vaccinations for certain occupational groups [12], could help reduce new HBV infections among adults in Georgia. Establishing mechanisms for monitoring hepatitis B immunisation coverage among adults would allow more accurate assessment of vaccine uptake.

Although screening programmes for certain groups (i. e. pregnant women, blood donors, military personnel, patients with hepatitis C) are in place, only a small proportion (13.5%) of our participants reported ever having been tested for HBV, and most HBsAg-positive persons in the survey had not previously been diagnosed. Therefore, hepatitis B diagnostic and treatment services across Georgia need to be expanded. Georgia could leverage the existing surveillance and clinical infrastructure of the hepatitis C elimination programme to establish a national HBV screening programme [18]. Further, expansion of proven approaches to integrated care (HIV, tuberculosis and harm reduction services) could help engage hard-to-reach population groups at high risk of hepatitis B [42-44].

Identification of patients needing treatment for HBV requires complex work-up [30], posing additional challenges to hepatitis B elimination. The survey suggested that a substantial number of persons in Georgia have HBV viral load levels that meet the current WHO criteria for treatment [30]. However, access to diagnostic services to help identify those in need of treatment varies across the country, and availability of antiviral treatment for hepatitis B is currently limited. There is no government-funded programme or private insurance which covers hepatitis B treatment. Removing financial barriers, ensuring availability of affordable antivirals and implementing effective linkages to a cascade of care for hepatitis B are essential for allowing equitable access to treatment for all eligible persons with chronic HBV infection in Georgia.

Only history of injecting drugs and inconsistent use of condoms were significant in multivariate analysis of risk factors for HBV infection. Associations with some other well-known risk factors for hepatitis B were not significant or were significant in bivariate analysis only, likely due to small numbers in the analysis subgroups. Notably, similar to the previous survey [6], only a small proportion of HBV-infected participants reported significantly associated risk factors, suggesting that targeted studies are needed to better elucidate risk factors for HBV infection in Georgia and tailor interventions to prevent HBV transmission among risk groups.

Awareness about hepatitis B among the adult population is limited. Communication campaigns on hepatitis B-related issues through sources of information most trusted by residents would help increase population awareness and generate demand for hepatitis B services. Along with the general population, communication efforts should specifically target healthcare providers and hard-to-reach groups.

The survey had certain limitations. Small numbers of HBV-infected participants precluded detailed analysis of HBV epidemiology among children and limited the statistical power of risk factor analysis among adults. Therefore, population-based cross-sectional design is probably not the optimal one for full elucidation of risk factors for HBV infection. Stigma may have limited the reporting of some risk factors (e.g. injection drug use, certain sexual practices, incarceration). Reliance on participant recall due to the lack of hepatitis B immunisation records for adults could have resulted in underestimation of vaccination levels. We were unable to conduct overall analysis of children’s hepatitis B vaccination levels due to the limited availability and low quality of historic immunisation records for children included in the survey (described in detail in the Supplement). In addition, at the time of writing this manuscript, specimens from the serosurvey participants had not been tested for hepatitis D virus. The implementation of the survey during the COVID-19 pandemic necessitated that field teams adhered to additional infection prevention measures. Otherwise, the pandemic did not negatively impact survey implementation as judged by the high participation rate.

## Conclusion

Georgia has achieved remarkable success with hepatitis B vaccination among children, but attention is needed on the burden of chronic HBV infection among adults. Georgia has demonstrated commitment to the elimination of hepatitis B through its inclusion in the National Strategy for Viral Hepatitis Elimination for 2021–2025. In addition, use of the existing framework and infrastructure of the hepatitis C elimination programme can help accelerate progress toward hepatitis B elimination. With near absence of the disease among children, focusing efforts on hepatitis B screening, treatment and preventive interventions among adults, along with sustaining high routine infant immunisation coverage, and leveraging synergies with the hepatitis C programme could help eliminate hepatitis B as a public health threat in Georgia by 2030. Lessons learned from the unique experiences of viral hepatitis elimination efforts in Georgia could aid other middle-income countries in the European Region and elsewhere to implement similar programmes.

## Supplementary Data

187000 Supplement
