# Supplementary material for: Toward reaching hepatitis B goals: hepatitis B epidemiology and the impact of two decades of vaccination, Georgia, 2021
Source: Euro Surveill. 2023 Jul 27;28(30):2200837. doi: 10.2807/1560-7917.ES.2023.28.30.2200837 (PMC10375835; doi:10.2807/1560-7917.ES.2023.28.30.2200837)
Supplement: Supplement [file 22-00837_KHETSURIANI_Supplement.pdf]

Toward reaching hepatitis B goals: hepatitis B epidemiology and the impact of two decades of vaccination, Georgia, 2021

Supplementary material

This supplementary material is hosted by Eurosurveillance as supporting information alongside the article "Toward reaching hepatitis B goals: hepatitis B epidemiology and the impact of two decades of vaccination, Georgia, 2021", on behalf of the authors, who remain responsible for the accuracy and appropriateness of the content. The same standards for ethics, copyright, attributions and permissions as for the article apply. Supplements are not edited by Eurosurveillance and the journal is not responsible for the maintenance of any links or email addresses provided therein.

Supplement 1. Nationwide serosurvey for COVID-19, hepatitis B and hepatitis C, Georgia, 2021: survey design and analysis

Sample design and size

We conducted a cross-sectional nationwide household survey using a stratified, multi-stage cluster design. The serosurvey was designed to provide estimates of the national and regional seroprevalence of SARS-CoV-2, hepatitis C virus and hepatitis B virus (HBV) infections in Georgia among children aged 5–17 years and adults aged ≥18 years. Participant enrolment took place during June–October 2021.

The survey sample size calculations for adults were based on the COVID-19 component, assuming 10% expected seroprevalence of anti-SARS-CoV-2 antibodies, ±1.1% margin of error, 95% confidence level, design effect (DE) 2.0 and 70% response rate, resulting in 8,010 persons (one adult per household). Based on the Georgia's National Statistics Agency (GEOSTAT) projections from the 2014 census data, it was assessed that approximately 32% of households would have at least one child of eligible age, resulting in approximately 2,560 expected households with children aged 5–17 years.

The sample size requirement for children was determined based on the 0.5% European regional Hepatitis B target for HBsAg prevalence and maximum 1.0% upper limit of the estimate considered acceptable by the European Regional Hepatitis B Working Group criteria for validation of the achievement of the regional target. Assuming 0.4% expected HBsAg prevalence (i.e. close to but below the regional target), precision level of ±0.35% (resulting in the 0.75% upper limit of prevalence estimate), design effect of 1.3, and 70% response rate, the sample size required for children was estimated as 2,320. This number was below the expected number of children in surveyed households (N=2,560). Therefore, sampling of 8,010 households was considered sufficient for estimating HBsAg prevalence among children as well.

However, due to the initial low response rate (60%) among children during the initial enrolment in June-August, an additional 1,880 households within originally selected clusters were approached for child enrolment. [1]Additional enrolment was done using the original methodology in 50 randomly selected clusters in four strata (capital city of Tbilisi, and Achara, Kakheti, and Kvemo Kartli regions) which had <60% response rate or were under-sampled during the initial allocation of sample, resulting in a total of 9,890 households approached for child enrolment.

[1] We opted for increasing size of existing clusters and taking the increased cluster size into account during analysis, because adding new clusters to the sample selected based on PPS was not feasible without compromising the integrity of the sample.

Participant selection and enrolment

The sampling frame was the general population of Georgia aged $\geq$5 years. A primary sampling unit (cluster) was defined as an enumeration area (EA) from the most recent, 2014 census. A total of 267 clusters were selected with probability proportionate to size from the list of all EAs. Within each selected cluster, 30 households were chosen systematically, using a skip pattern. In the 50 clusters with additional child enrolment, an additional 30–45 households were chosen using the identical method and approached for child enrolment only. Finally, one adult aged $\geq$18 years and one child aged 5–17 years (in households with at least one child of eligible age) per household were selected using Kish tables.

The sample was stratified into 10 strata, which included all regions and the capital city of Tbilisi. The Autonomous Republic of Abkhazia and a section of the Shida Kartli region (South Ossetia), currently outside Georgian government control, were excluded. In addition, Racha-Lechkhumi-Kvemo Svaneti region with small population (n=30,778), was combined in one stratum with the adjacent Imereti region.

Participants were enrolled after obtaining informed consent from participants aged ≥18 years or from parents/caregivers of children aged 5–17 years; assent was obtained from children aged 7–17 years. Persons with mental illness precluding consent and any participants who could not give blood because of severe illness or hemophilia were excluded.

Because the serosurvey was implemented during the COVID-19 pandemic, field teams followed strict infection control measures recommended by the Georgian government at the time. Survey questionnaire was administered by trained field staff in face-to-face interview with participants or their parents/caregivers (for children). The responses were recorded electronically using tablets and uploaded to a cloud-based server (ODK). Each participant's questionnaire was labeled with a unique identifier (barcoded label) that was linked to their blood sample to maintain confidentiality and allow linking of laboratory results and notification to individuals testing positive. Test results were provided to participants within a maximum six months after sample collection. Hepatitis B virus (HBV)-infected individuals were counseled and referred to a local provider for linkage to care.

Questionnaire and immunization information

A survey questionnaire included demographics, clinical and behavioral history, potential exposures to risk factors for HBV infection, immunization status, and hepatitis B-related awareness and practices.

Participants aged 5–20 years (i.e. born in or after 2001) had been eligible for routine hepatitis B vaccination as infants. Hepatitis B vaccination information for adults was based on participant recall. For children, we requested home-based immunization records. If records were not available at home, we searched the National Immunization Registry, except when a parent stated that the child was unvaccinated. For all HBV-infected children, hepatitis B immunization history obtained through the immunization registry search was verified with respective healthcare facilities. We did not conduct initially planned analysis of overall hepatitis B vaccination levels among enrolled children because historic hepatitis B immunization data for children's age groups included in the survey, were found to be limited and of inconsistent quality[2].

Statistical Analysis

To produce nationally representative estimates, results were weighted at cluster, household, and individual levels, and estimates were adjusted by sex, age, and geographic distribution using 2014 census data. Weights were calculated separately for adults and children, taking into account additional

[2] Traditionally, immunization records in Georgia are stored at healthcare facilities and, in recent years, immunization data are entered into the National Immunization Registry initiated in 2015-2016. When survey teams visited houses, only 22 of 1473 children had immunization records at home and another 12 children were unvaccinated per parental recall. The National Immunization Registry search conducted for the remaining 1439 children, located the records for only 830 of them and often were incomplete. The reason for low completeness was that the survey age cohorts were born before the Registry was initiated. Although children's healthcare providers were instructed to enter historic data on vaccinations given in earlier years, this was apparently done inconsistently, resulting in many missing or partially completed records which made it difficult to determine if the missing vaccine doses were not given or not entered.

enrolment for children. Primary outcome measures for seroprevalence were adjusted proportions and 95% confidence intervals (95% CI) were calculated.

Seroprevalence estimates by age group, sex, and region were calculated. Potential risk factors for anti-HBc-positivity (i.e. risk factors for acquiring HBV infection) were analyzed in bivariate and multivariate analysis. Chi-square test was used in bivariate analysis, with p values <0.05 considered significant. Variables associated with anti-HBc positivity in bivariate analysis were included in the multivariable regression model and adjusted odds ratios (OR) and 95% CIs were calculated.

In the analysis, anti-HBc-negative persons were considered unexposed/uninfected with HBV. Among anti-HBc-positives, HBsAg-positive participants were considered having chronic HBV infection[3], while HBsAg-negative persons were considered having resolved HBV infection [1]. The HBV viral load levels were categorized as <2,000 IU/mL, 2,000-19,000 IU/mL and ≥20,000 IU/mL [2].

Prevalence estimates for adult participants were compared with those from the baseline 2015 serosurvey which had comparable design [3,4]. The 2015 survey was a nationwide household survey using the same sampling frame and similar multi-stage cluster survey design as the current survey and targeted 7,000 adults aged ≥18 years at the time. Among them, 6,007 persons were tested for anti-HBc and HBsAg. For direct comparability, the 2015 survey data were re-analyzed to include age groups similar to the ones used in the analysis of the current survey data. Additionally, 6 years were added to ages of participants of the 2015 serosurvey to adjust to their ages in 2021. All analysis was performed in SAS version 9.4 (Cary, North Carolina, USA).

References

1. WHO. Documenting the Impact of Hepatitis B Immunization: best practices for conducting a serosurvey. Available at: http://apps.who.int/iris/bitstream/handle/10665/70808/WHO_IVB_11.08_eng.pdf;sequence=1
2. WHO. Guidelines for the prevention, care and treatment of persons with chronic hepatitis B infection. WHO, 2015. https://apps.who.int/iris/bitstream/handle/10665/154590/9789241549059_eng.pdf?sequence=1
3. Kasradze A, Shadaker S, Kuchuloria T, et al. The burden and epidemiology of hepatitis B and hepatitis D in Georgia: findings from the national seroprevalence survey. Public Health. 2020;185:341-47.
4. Hagan LM, Kasradze A, Salyer SJ, et al.. Hepatitis C prevalence and risk factors in Georgia, 2015: setting a baseline for elimination. BMC Public Health. 2019;19(Suppl 3):480.

[3] The probability of encountering active acute HBV infection in a cross-sectional survey in a setting with low incidence of HBV is considered very low [1].

Supplement 2. Hepatitis B-related awareness among survey participants, Georgia, 2021

Questions	Weighted % (95% CI)
Have you ever heard of the hepatitis B virus?	
Yes	34.9 (32.6–37.4)
How do you think hepatitis B virus is transmitted?	
Droplets	14.8 (12.3–17.8)
Food	6.2 (4.4–8.7)
Blood	87.9 (85.3–90.1)
Sexual contact	45.5 (41.2–49.9)
From mother to child	22.5 (18.9–26.6)
Handshake with infected person	7.1 (5.5–9.1)
Sharing household objects	41.9 (37.3–46.7)
Sharing needles or syringes	45.9 (40.7–51.0)
Touching items in public places	5.6 (3.8–8.1)
Don't know	20.4 (17.5–23.6)
Correctly identified ≥1 transmission routes and gave no incorrect answers	59.9 (55.9–63.8)
Mix of correct and incorrect answers	17.5 (14.8–20.5)
All answers incorrect or did not know	22.6 (19.6–26.0)
Is it possible to have the hepatitis B virus but not have any symptoms?	
Yes	50.1 (46.2–54.1)
Are there medications available to treat hepatitis B virus infections?	
Yes	46.0 (42.4–49.6)
What can you do to help prevent hepatitis B virus infection?	
Vaccination	21.6 (18.9–24.7)
Condom use	45.0 (40.9–49.3)
Avoid sharing needles	7.8 (6.1–9.9)
Wash hands thoroughly	43.8 (39.9–47.8)
Avoid unsterile/used medical devices	27.4 (24.0–31.1)
Don't know	21.6 (18.9–24.7)
Correctly identified ≥1 prevention strategy and gave no incorrect answers	64.1 (60.5–67.4)
Mix of correct and incorrect answers	7.6 (5.9–9.7)
All answers incorrect or did not know	28.3 (25.0–32.0)
Where do you get information about health that you trust?	
Family, friends, neighbors, colleagues	33.9 (31.5–36.5)
Special medical literature	4.9 (4.1–5.8)
Newspapers and magazines	2.6 (2.0–3.4)
Radio	1.4 (1.0–1.9)
TV	51.4 (48.6–54.3)
Internet	39.2 (37.0–41.3)
Billboards	1.2 (0.9–1.8)
Brochures, fliers, printed material	2.8 (2.1–3.6)
Doctors, other healthcare workers	47.9 (44.6–51.2)
Pharmacists	4.4 (3.5–5.6)
Don't know/remember	2.6 (2.0–3.4)
None of the above	1.1 (0.8–1.5)

All participants were asked questions about having heard of hepatitis B and about trusted sources of information; respondents for other questions were those who have heard of hepatitis B
